# Hypnosis-based psychodynamic treatment in ALS: a longitudinal study on patients and their caregivers

**DOI:** 10.3389/fpsyg.2015.00822

**Published:** 2015-06-16

**Authors:** Johann R. Kleinbub, Arianna Palmieri, Alice Broggio, Francesco Pagnini, Enrico Benelli, Marco Sambin, Gianni Sorarù

**Affiliations:** ^1^Department of Philosophy, Sociology, Pedagogy and Applied Psychology, University of PadovaPadova, Italy; ^2^Center for Cognitive Neuroscience, University of PadovaPadova, Italy; ^3^Department of Psychology, Catholic University of MilanMilan, Italy; ^4^Department of Neurosciences, University of PadovaPadova, Italy

**Keywords:** amyotrophic lateral sclerosis (ALS), hypnosis, anxiety, depression, quality of life, defense mechanisms, ALSFRS-r, psychodynamic intervention

## Abstract

**Background:** Evidence of psychological treatment efficacy is strongly needed in ALS, particularly regarding long-term effects.

**Methods:** Fifteen patients participated in a hypnosis treatment and self-hypnosis training protocol after an in-depth psychological and neurological evaluation. Patients' primary caregivers and 15 one-by-one matched control patients were considered in the study. Measurements of anxiety, depression and quality of life (QoL) were collected at the baseline, post-treatment, and after 3 and 6 months from the intervention. Bayesian linear mixed-models were used to evaluate the impact of treatment and defense style on patients' anxiety, depression, QoL, and functional impairment (ALSFRS-r), as well as on caregivers' anxiety and depression.

**Results:** The statistical analyses revealed an improvement in psychological variables' scores immediately after the treatment. Amelioration in patients' and caregivers' anxiety as well as caregivers' depression, were found to persist at 3 and 6 months follow-ups. The observed massive use of primitive defense mechanisms was found to have a reliable and constant buffer effect on psychopathological symptoms in both patients and caregivers. Notably, treated patients decline in ALSFRS-r score was observed to be slower than that of control group's patients.

**Discussion:** Our brief psychodynamic hypnosis-based treatment showed efficacy both at psychological and physical levels in patients with ALS, and was indirectly associated to long-lasting benefits in caregivers. The implications of peculiar psychodynamic factors and mind-body techniques are discussed. Future directions should be oriented toward a convergence of our results and further psychological interventions, in order to delineate clinical best practices for ALS.

## Introduction

Amyotrophic Lateral Sclerosis (ALS) erodes patients' personal autonomy and their own freedom in many dimensions of existence. This relentless syndrome mainly affecting the voluntary motor system is characterized by increasing weakness, spasticity, and dysarthric speech. Potential executive dysfunction has been described, susceptibly to demographic variables (Palmieri et al., 2014), in a relevant proportion of cases (Abrahams, 2013). The disease is also featured by secondary symptoms such as pain (Pagnini et al., 2012a), sleep disorders (Blackhall, 2012), emotional lability (Palmieri et al., 2009) and fasciculations (Rana et al., 2009), that generate from mild to severe discomfort in everyday living. In the late stages of the disease, progressing muscular degeneration can evolve in a “locked-in” state in which conscious patients becomes progressively paralyzed and voiceless (Borasio et al., 2001). Death usually occurs due to respiratory failure within 3–4 years from the onset, unless life is protracted by tracheostomy and long term-mechanical ventilation (LTMV). In this case, the patient's life can be extended 10 years or more after the onset in locked-in state (Vianello et al., 2011).

Among the wide range of reactions in which a human being could react to the diagnosis of ALS and its devastating consequences, such as apathy, denial (Ferro et al., 1987) stoicism (Rabkin et al., 2000), resentment, hate, hope (Oster and Pagnini, 2012), or suicidal ideation (Palmieri et al., 2010a), anxiety and depression are probably the most expected features. While their real prevalence is still uncertain (Pagnini, 2013) most studies report values around 30% for anxiety and 40% for depression with some studies reporting values up to 70% for both (Kurt et al., 2007; Wicks et al., 2007; Palmieri et al., 2009; Pagnini, 2013).

Quality of life (QoL) is a further theoretical and clinical issue relevant in ALS that could reasonably be directly associated with physical decline. Several studies suggest, conversely, that ALS patients' QoL is independent of physical impairment (Simmons et al., 2000; Palmieri et al., 2010b; Pagnini, 2013), a phenomenon known as “disability paradox” (Carr and Higginson, 2001), although this relationship is still unclear and motor disability or ALS physical consequences are likely to play a crucial role in determining QoL (Ganzini et al., 1999; Lo Coco et al., 2005). QoL has also been negatively associated to psychological variables such as suffering, sense of burden and hopelessness and positively related mainly to spirituality (McLeod and Clarke, 2007; Pagnini et al., 2011), caregiver relations (Chiò et al., 2004), and mindful attitude (i.e., not evaluating a situation or a context only through previous categorizations, but actively making new distinctions by assuming multiple viewpoints and perspectives; Pagnini et al., 2014a). Fegg et al. (2005) and Roach et al. (2009), highlight a usually undervalued viewpoint, i.e., personality and personal attitudes strongly modulate and shape in a unique manner the possible reactions to ALS.

It is extremely complex to disentangle the specific role of psychological variables, including anxiety, depression, and subjectively perceived QoL from peculiar individual characteristics and physical impairment. It is common clinical experience that these dimensions, in ALS, are very tightly interconnected in a dynamic and reciprocally causative way, forming a circular system not yet thoroughly studied.

Indeed, the great physical burden induced by ALS easily implies negative psychological reactions according to individual traits and attitudes, which, in turn, could weigh on the physical level. This latter aspect has an enormous importance in a disease that substantially lacks medical treatment (Beghi et al., 2011) and reliable physical prognostic factors (Creemers et al., 2014).

The evidence for the strong relation between psychological status and physical outcome in ALS was first provided by a seminal, cross-sectional longitudinal study by McDonald et al. (1994), who found that patients with psychological distress showed a significantly greater risk of mortality than those with higher psychological well-being. Similarly, Johnston et al. (1999) and Krampe et al. (2008) found that mood and personality traits can strongly influence the survival rate of ALS patients. Recently, Pagnini et al. (2014a) showed on a large sample of people with ALS that a mindful attitude attenuates the ALS progression.

It is easy to imagine how being affected by ALS can have devastating psychological impact not only on patients but also on their families, and mostly on their primary caregivers, which are as well at risk of developing depressive and anxiety reactions (Pagnini et al., 2010, 2011; Cipolletta and Amicucci, 2014). It was first reported, in the seminal results by Chiò and his team (Gauthier et al., 2007; Vignola et al., 2008), that whilst in patients depression, anxiety and QoL remained substantially steady, in caregivers there was a significant decrease of Qol and an increase of anxiety and depression along disease progression.

However, notwithstanding the potentially great relevance of the topic, scientific literature quite completely neglected research on the efficacy of psychological interventions. Pagnini et al. (2012c) recently called the scientific community via ALS leading journal to fill this void, highlighting the importance of developing “best practices” for the improvement of QoL and the reduction of psychological distress in ALS patients.

As a first response to this call, Palmieri et al. (2013) published a pilot study on psychodynamic-oriented psychological treatment on ALS, showing the efficacy of a hypnosis intervention together with a self-hypnosis training protocol. The efficacy was proved by measuring pre-post levels of anxiety, depression, QoL, and perceived changes of the aforementioned secondary physical symptoms. Improvements in caregivers' anxiety and depression, probably as a consequence of patients' psychological and perceived physical symptomatology improvement, were also observed. In a further, recent study by Averill et al. (2013), a structural emotional disclosure intervention was proposed to patients with ALS. Authors found their treatment to improve psychological well-being, but argued that their kind of intervention may only be helpful for ALS patients who were unable to express emotions or who had ambivalent attitude about expressing emotion to others. Díaz et al. (2014) demonstrated the benefit, in terms of both anxiety and depression levels, of an integrated intervention based on counseling and cognitive behavioral therapy.

Similarly, Pagnini and colleagues are collecting data from a randomized controlled trial to evaluate the effects of an ALS-specific meditation training (Pagnini et al., 2014b) on well-being of people with ALS and their caregivers.

Thus, the few and recent research efforts on psychological intervention on ALS displayed very encouraging results, nonetheless they are deeply different in terms of adopted psychological approach, methodology employed and measures used to evaluate the change. Moreover, peculiar individual characteristics that may mediate the change have never been specifically assessed, and no long-term disease progression implications have been studied, so far, in correspondence to specific psychological treatment.

Our aim is to extend our previous (Palmieri et al., 2013) hypnosis-based intervention to a broader ALS group, investigating the longitudinal, long-term effects of intervention on patients and their caregivers, and taking into account the impact on disease progression as well as the influence of individual aspects.

In the direction of this latter goal, we privileged a psychodynamic approach to highlight personality features that could mediate the adaptation to the disease, specifically by focusing on defense mechanisms, which offer an interesting insight into the ego's psychological functioning.

In synthesis, we studied the long-term effects of a brief psychodynamic hypnosis-based intervention on patients with ALS, mainly in terms of their psychopathological and physical symptoms as well as its resonance on caregivers. As a first hypothesis, in line with previous data (Palmieri et al., 2013), we expected positive changes on patients' anxiety, depression, and QoL and that these changes, as measured after the psychodynamic hypnosis-based intervention, would keep stable after 3 and 6 months. Secondly, on the basis of previous results linking psychological well-being and disease progression (McDonald et al., 1994; Johnston et al., 1999; Krampe et al., 2008; Pagnini et al., 2014a), we investigated the possibility of a positive physical effect on disease progression in the treatment group in comparison to the control group. Thirdly, we further hypothesized that these effects of the treatment on patients would also have, as well, an indirect beneficial impact on caregivers' anxiety and depression levels.

Finally, we hypothesized that intrapsychic dynamic factors, such as defense mechanisms, could also represent a starting point in order to take into account both the strictly nomothetic and the idiographic perspectives as complementary (Salvatore and Valsiner, 2010) since the uniqueness of psychological phenomena requires characterizing the dynamics of the individual cases while striving for generalization.

## Methods

### Participants

Fifteen consecutive volunteering patients with ALS—as members of the treatment group—their respective primary caregivers, and 15 patients with ALS—as control subjects—were recruited via the Motor Neuron Disease Center of the University of Padova Hospital. In synthesis, a total of 45 individuals participated in the study. Participants were informed about the study's purpose and methods and signed informed consent to the study protocol, which was approved by the Ethical Committee of the University of Padova, and carried out in accordance with the principles of the Helsinki Declaration as revised in 1983.

The patients in the treatment group were 7 males and 8 females, with ages ranging from 43 to 73 years (*M* = 55.3, *SD* = 8.72); the mean time since diagnosis ranged between 2 and 37 months (*M* = 14.79; *SD* = 11.05); 11 patients had limbic onset, while 4 had bulbar onset; 4 patients were treated with low dosage antidepressant, 1 patient with anxiolytic, and 11 patients were treated with Rilutek. One of the patients died 6 months after the recruitment, thus only available data was analyzed for this patient. Their caregivers were 12 spouses (5 males, 7 females) and 3 daughters.

The patients in the control group were selected among the available Motor Neuron Disease Centre's longitudinal dataset and were matched for functional disability (ALS-FRS-r; Cedarbaum et al., 1999), age, gender, onset site, time from onset, absence of any cognitive impairment, and were not receiving psychological assistance during the observational time range.

Inclusion criteria for all patients were to have sporadic ALS fulfilling the revised El Escorial criteria for clinically probable or definite ALS (Brooks et al., 2000). Exclusion criteria for patients were the presence of significant neuropsychological dysfunctions or alterations if compared to normative data, while for both patients and caregivers, the presence of concomitant psychiatric or neurological illness, and current use of high-dose psychoactive medications would lead to exclusion. No participant was excluded by these means.

### Measures

To assess defense mechanism profile, and thus infer developmental levels in a psychodynamic perspective, we used the Defense Style Questionnaire (DSQ; Bond et al., 1983). The DSQ is a widely used self-report measure of empirically derived groupings of defense mechanism ranked on an adaptive hierarchy. The questionnaire consists of 40 items on a nine point Likert scale, ranging from “Completely Agree” to “Completely Disagree.” Four defensive styles labeled maladaptive, image-distorting, self-sacrifice, and adaptive are evaluated. Authors of the Italian validation of the questionnaire (San Martini et al., 2004) suggested mostly using the maladaptive subscale since it has the most items and shows higher reliability.

Trance depth and hypnotizability were assessed through the Hypnoidal State Score (HSS) of the Phenomenology of Consciousness Inventory (PCI; Pekala and Levine, 1982; Pekala, 1991). The PCI is a 53-item self-report inventory that maps 12 major and 14 minor dimensions of subjective experience. In addition to quantifying the experience of being hypnotized, this measure describes various aspects of subjective experience, such as imagery, affects, internal dialog, and alterations in awareness, which were used to tailor subsequent hypnotic interventions according to the patients' features.

The Hospital Anxiety and Depression Scale (HADS; Zigmond and Snaith, 1983) is a broadly used and reliable (Bjelland et al., 2002) self-report measure for caseness and symptom severity of anxiety disorders and depression. It consists of two subscales of 7 items each ranging from 0 to 3: HADS-A, assessing anxiety and HADS-D for depressive symptoms. Among the wide range of generic depression scales employed in ALS context, the HADS items content seem to be less prone to be distorted by ALS' somatic impairments compared to other common depression measures (Pagnini et al., 2014c). Furthermore, a motor neuron disease's specific version of the HADS has been developed and quantitatively confirmed using Rasch analysis in a study by Gibbons et al. (2011), providing revised scoring and cut-offs that were used for patients in the present study.

The Amyotrophic Lateral Sclerosis Specific Quality of Life—revised (ALSSQOL-r; Felgoise et al., 2009; Pagnini and Simmons, 2010), was used to assess different dimensions of health-related QoL. The ALSSQOL-r is a 46 item disease-specific questionnaire using a 0–10 point Likert scale, with 0 being the least desirable situation, and 10 being the most desirable. It consists of six subscales: Negative Emotion, Interaction with People and the Environment, Intimacy, Religiosity, Physical Symptoms, and Bulbar Function; a Total score, including all the items can be computed and was used in analyses. The ALSSQOL-r was chosen since it provides insights into psychological aspects, spirituality and social issues associated with the disease (Simmons et al., 2006).

Functional impairment owing to ALS was evaluated using the ALS-Functional Rating Scale Revised (ALS-FRS-R; Cedarbaum et al., 1999), which represents the gold standard for neurological evaluation of physical decline imposed by the disease. It consists of 12 items with scores ranging from 0 to 4, with a maximum achievable score of 48 (0 = total disability, 48 = normal) that measures bulbar, upper-extremity, lower-extremity, and respiratory functions.

### Procedure and intervention

Patients underwent complete neurological examination and exhaustive neurocognitive assessment on the same day, about 2 weeks before the psychological treatment, respectively performed by a trained expert neurologist and neuropsychologist with long-term experience with ALS. These evaluations were performed as part of the standardized diagnostic routine at the Motor Neuron Disease Center.

A few days after examination, fully eligible patients of the treatment group underwent a clinical interview performed by a psychotherapist experienced in ALS. This interview was aimed to assess patients' defensive styles (through the DSQ questionnaire) and personality characteristics, crucial in shaping the psychological intervention planning. In this context, patients were encouraged to share possible doubts or curiosity about theoretical premises and expected outcomes, in order to fully inform them about the study and to build clinical alliance. An analogous clinical interview was performed by psychotherapists with caregivers.

The intervention protocol consisted of 4 weekly domiciliary sessions of hypnosis treatment. Each session was conducted by a trained operator in Ericksonian hypnosis accompanied by a further psychologist who administered all the questionnaires and did not remain in the same room during treatment. Each hypnotic session consisted of three phases: the first one was a standardized hypnotic induction based on well-established ideodynamic techniques (Bernheim, 1880) and focused on mind-body relaxation, lasting about 20 min. The second, core phase, lasting from 30 to 45 min, consisted in the administration of therapeutic metaphors, guided visual imagery, and direct and indirect suggestions individually tailored on the basis of the needs of each patient. The conclusive phase of each session consisted of anchoring suggestions aimed to teach self-hypnosis, followed by a slow, guided return to a completely awake state of full consciousness, lasting about 10 min.

While the individual suggestions and metaphors were specifically chosen on a per-patient basis, according to their evolving clinical condition, personality and history, the four sessions were based on fixed themes, common to every patient. In the first session, labeled “safe place,” the suggestions and imagery were oriented to a safe and quiet place where the patient could allow his own body and mind to rest and recover in deep relaxation. In the second session, labeled “awareness,” the suggestions were focused on developing awareness of individual thoughts, emotions and soothing body's perceptions like the breathing rate. In the third session, labeled “life chain,” the suggestions were directed to the change of time focus, and to images of the patients' own familial generations. The fourth and final session, labeled “perceptive,” was focused on various imagined perceptions associated to positive emotions and metaphors. After each hypnosis session, perceived trance depth was assessed through the PCI.

The first phase standardized induction, common to all sessions and for all patients, was recorded on an audio compact disk and was left for each patient to listen to in order to enhance self-hypnosis. Patients were encouraged to practice self-hypnosis at least once every day. The whole procedure, including self-hypnosis training, was partially inspired by the protocol by Jensen et al. (2009), Jensen (2011) successfully applied on patients affected by multiple sclerosis in terms of increased well-being.

In synthesis, before the first session and in each follow-up, depression, anxiety levels (by means of HADS) and quality of life (by means of ALSSQOL-r) were assessed. HADS was administered also to caregivers on these occasions. DSQ was assessed only at the preliminary psychological interview, while PCI was administered after each hypnotic session, as it is a specific evaluation of the hypnotic experience.

Information about the presence of the most common ALS secondary symptoms, i.e., pain, sleep disorders, emotional lability, and fasciculations, was verbally collected by the psychologist at the beginning and at the end of treatment.

From an experimental design perspective, outcome variables were anxiety, depression (both for the patients being treated and their caregivers), QoL, and functional disability. These were measured before the intervention (T0), after the last domiciliary session (T1) and on two follow-ups after three (T2), and six (T3) months from the treatment. Since physical functioning alterations, as assessed by ALSFR-r are not appreciable after just 1 month, measurements were collected only at T0, T2, and T3 for both treatment and control patient groups.

### Statistical analysis

Bayesian mixed-models methods were chosen because they offer distinct advantages over classic NHST approaches, such as a more straightforward interpretation of the results (Dienes, 2011), a better handling of outliers and multiple comparisons (Gelman et al., 2012; Aguinis et al., 2013). Furthermore, relevantly to our design, this approach is more robust with small samples and relies on fewer and less strict assumptions on the data (Hoijtink et al., 2008). For a thorough review of the advantages of Bayesian inference in psychological research, see Wagenmakers et al. (2008).

All the analyses were performed with R statistical software, version 3.1.1 (R Core Team, 2014) through the “BayesFactor” package v.0.9.8 (Morey et al., 2014), using non-informative priors. All the Markov Chain Monte Carlo simulations were run with 100,000 iterations.

#### Regression analyses

For each of our dependent variables, i.e., HADS-A and HADS-D, ALSFR-r and ALSSQOL-r we performed a Bayesian linear mixed-model regression analysis, considering subjects as a random factor (Id) and observation time-points (labeled “Time”), months since diagnosis, HSS score, DSQ scores, psychopharmacological drugs usage, self-hypnosis practice, and their interactions as fixed factors. We computed a Bayes Factor (BF) of every possible model from our factors, against the simplest model with only the Id random factor. We then chose the most parsimonious model that was not less supported by data than 3 times the one with the highest overall evidence. This cut-off was chosen since smaller BF are described in literature (Jeffreys, 1961) as indicating a “barely worth mentioning,” and not “substantial” evidence for the model. The models selected through this statistical approach were also valued in the light of their theoretical soundness against similarly evident models. These two criteria always converged in the choosing of the best models. Finally, we computed the posterior distributions, quantifying uncertainty, for each of the parameters of the chosen models.

#### Groups comparison

The comparison between the treatment and control groups were performed through Bayesian hypothesis testing (Rouder et al., 2009; Kruschke and Meredith, 2014). We estimated the difference in means between the two groups ALSFRS-r decline (T3 minus T0 scores) and computed its posterior probability distribution to assess uncertainty. From this distribution, the mean credible value was taken as the best guess of the actual difference and the 95% Highest Density Interval (HDI) as the range where the actual difference is with 95% credibility.

## Results

### Patients and caregiver psychological measurement

Before treatment (T0), patients had a mean HADS-A score of 7.60 (*SD* = 3.94), 3 patients' score was in the range for “borderline” cases of anxiety, and 6 patients' score was higher than the “case level” cut-off. After the 6 months follow-up (T3), the mean score was 4.50 (*SD* = 2.62) and only 1 patient's score was in the range for “borderline” cases of anxiety, and 1 patient's score was higher than the “case level” cut-off.

Before treatment (T0), patients had a mean HADS-D score of 3.27 (*SD* = 1.98), 5 patients' score was in the range for “borderline” cases for depression, while no patients' score was higher than the “case level” cut-off. After the 6 months follow-up (T3), the mean score was 2.64 (*SD* = 1.91) and 3 patient's score was in the range for “borderline” cases for depression, and none of the patient's score was higher than the “case level” cut-off.

**Figure 1:**
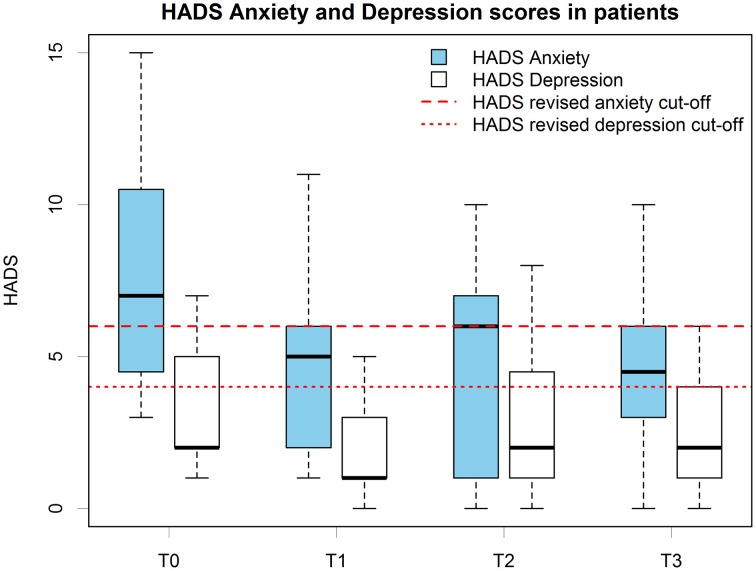
**Patients' HADS anxiety and depression scores boxplots at all time points**. Scoring and cut-offs are based on the revised version for MND patients.

HADS scores for the treatment group are presented in Figure 1. The deceased patient did not have an anxiety or depression score over the cut-offs at any measured time.

**Figure 2:**
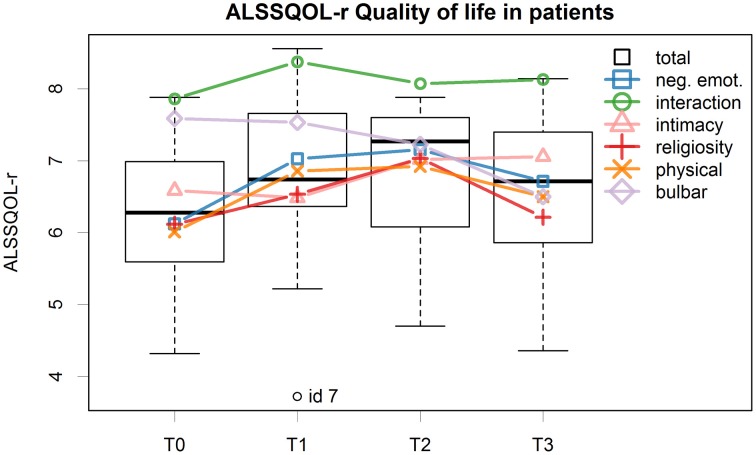
**Patients' ALSSQOL-r total scores for all time points are reported in the boxplots, the lines represents the individual subscales**.

ALSSQOL-r scores (Figure 2) were in line with the normative data for ALS patients (Felgoise et al., 2009; Pagnini and Simmons, 2010) with a mean total score of 6.83 (*SD* = 1.10) at T0 and of 7.07 (*SD* = 1.14) at T3.

Mean ALSFRS-r score at T0 was 33.13 (*SD* = 9.88) for the treatment group and 31.27 (*SD* = 3.90) for the control group; at T3 these mean scores declined to 31.00 (*SD* = 8.13) for the treatment group and to 23.00 (*SD* = 7.25) for the control group.

The DSQ scores, measured at baseline, showed a very high defensive behavior in treatment group's patients. Specifically the maladaptive (*M* = 6.90, *SD* = 1.04) and image-distorting (*M* = 6.73, *SD* = 0.74) subscales scores, assessing the use of less mature defense mechanisms, were found to be more than the double of the normative data scores for the Italian population (San Martini et al., 2004). Adaptive subscale scores (*M* = 3.84, *SD* = 1.36), in contrast, were comparable to the normative data. The mean scores for patients' and caregivers' individual defense mechanism are reported in Table 1.

**Table 1:** **Individual defense mechanisms' mean scores of patients and caregivers as assessed by Defense Style Questionnaire at baseline**.

**Defense style**	**Defense mechanisms**	**Patients**		**Caregivers**	
		***M***	***SD***	***M***	***SD***
Maladaptive	Acting out	6.80	1.70	6.19	1.60
	Undoing	6.40	1.84	6.69	1.91
	Passive aggressive	6.69	1.42	6.53	1.35
	Consumption	7.45	2.02	7.24	1.57
	Fantasy	7.29	2.46	4.50	2.69
	Projective identification	8.00	1.81	8.21	2.01
	Help-rejection	6.04	1.94	5.83	1.98
	Projection	7.56	0.91	7.00	1.09
	Regression	7.04	1.75	6.39	2.29
	Withdrawal	5.07	2.85	4.05	2.25
	Somatization	7.71	2.31	7.14	2.33
Image-distorting	Denial	6.17	1.70	6.05	1.29
	Isolation	5.88	1.36	4.66	1.73
	Omnipotence	7.62	1.04	7.23	1.22
	Splitting	7.24	1.52	5.86	1.87
Adaptive	Affiliation	6.40	1.39	5.11	2.56
	Anticipation	3.10	1.84	4.04	2.29
	Primitive idealization	5.32	3.12	5.14	2.70
	Task orientation	3.33	2.78	2.32	1.38
	Pseudo altruism	2.20	1.90	2.50	2.20
	Suppression	3.90	2.23	3.79	1.87
	Sublimation	5.47	3.38	4.14	3.11

For each patient, the average HSS score of the four hypnotic sessions was calculated. All the patients were able to successfully achieve an adequate trance depth: 5 out of 15 patients were able to enter a mild hypnoidal state (HSS between 3 and 5), and the remaining 10 patients a moderately hypnoidal state (HSS between 5 and 7).

Before treatment (T0), caregivers had a mean HADS-A score of 9.13 (*SD* = 3.72), 3 caregivers' score was in the range for “borderline” cases of anxiety, and 7 caregivers' score was higher than the “case level” cut-off. After the 6 months follow-up (T3), the mean score was 5.07 (*SD* = 3.67) and only 1 caregivers' score was in the range for “borderline” cases of anxiety, and 2 caregivers score was higher than the “case level” cut-off.

**Figure 3:**
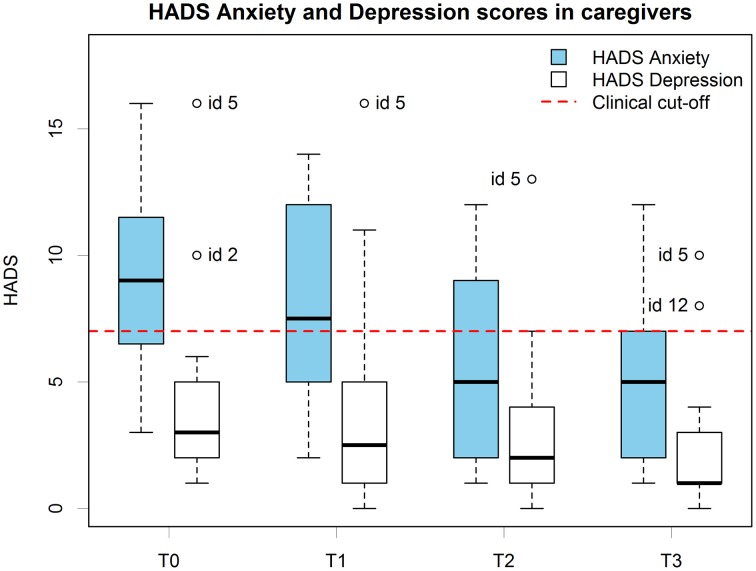
**Caregivers' HADS anxiety and depression scores boxplots at all time points**. Scoring and cut-offs are based on the original scale.

Before treatment (T0), caregivers had a mean HADS-D score of 4.60 (*SD* = 3.87), 1 caregiver's score was in the range for “borderline” cases for depression, while another 1 caregiver's score was higher than the “case level” cut-off. After the 6 months follow-up (T3), the mean score was 2.50 (SD = 3.03) and 2 caregivers' score was in the range for “borderline” cases for depression, and none of the caregivers score was higher than the “case level” cut-off. HADS scores for the caregivers group are presented in Figure 3.

### Psychological wellbeing of ALS patients: Longitudinal results

#### Anxiety

**Figure 4:**
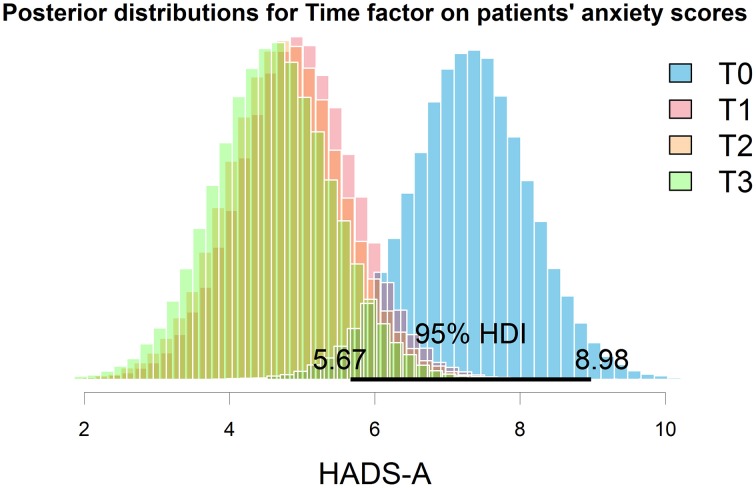
**Posterior distributions of anxiety means at the different time points**. The 95% HDI represents the area with the most credibility for the exact value. Graphical inspection clearly evidence the decrease in the HADS anxiety score after the treatment, and that this improvement was maintained in the further follow-ups.

The best model selected through the Bayesian linear regression showed a strong effect of the treatment (Time factor) and of the DSQ maladaptive style score but no interaction factors. None of the other considered moderators were present in the most plausible models. Figure 4, representing the posterior distributions of HADS-A means at the different time points for the selected model, shows that the reduction in anxiety scores after the treatment kept stable at T2 and T3.

**Figure 5:**
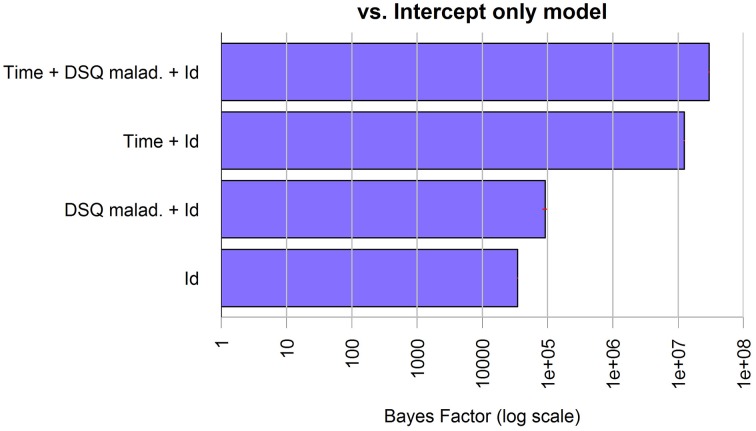
**Bayes Factors of the chosen model for anxiety in patients and its simpler nested models against the null model**. The bars, on logarithmic scale, represents the ratio of evidence between the different models. Thus, by visual comparison, the weight of each single factor can be inferred.

The model showed an overall good fit [BF against “Id only” model = 874, *R*^2^ = 0.79, Residual Standard Error (RSE) = 1.92, model parameters are reported in Table 2]; additional Bayes Factors are reported in Figure 5.

**Table 2:** **Posterior parameters for HADS-Anxiety best regression model in patients**.

**Parameter**	**Mean**	***SD***	***SE***
Intercept	5.404	0.717	0.002
T0	1.902	0.450	0.002
T1	−0.460	0.401	0.001
T2	−0.638	0.404	0.001
T3	−0.804	0.418	0.001
DSQ–maladaptive	−1.066	0.686	0.003
Sigma^2^	3.665	0.846	0.005

#### Depression

While the data actually showed a possible reduction in depression after the treatment, patients' depression scores at T2 increased again at pre-treatment levels (Figure 1). None of the considered factors in regression analysis showed a relevant linear effect on depression.

#### Quality of life

The best model for the ALSSQOL-R QoL total score showed a relevant, although small, effect of treatment. The score improved at T1 and T2, while at T3 decreased again toward the pre-treatment score. This trend of the Total score was similarly found in most subscales, with the exception of the “bulbar” subscale score, which showed a constant decline, and the “intimacy” subscale score which showed an amelioration only at T2 and kept stable at T3 (Figure 2).

The selected model, with QOL predicted by Time and Id, showed an overall good fit (BF against “Id only” model = 8.86, *R*^2^ = 0.82, RSE = 0.45, model parameters are reported in Table 3). None of the other considered moderators were present in the most plausible models.

**Table 3:** **Posterior parameters for ALS Specific Quality of Life-revised questionnaire best regression model for patients**.

**Parameter**	**Mean**	***SD***	***SE***
Intercept	6.579	0.269	0.001
T0	−0.232	0.097	<0.001
T1	0.162	0.093	<0.001
T2	0.167	0.093	<0.001
T3	−0.097	0.093	<0.001
Sigma^2^	0.202	0.047	<0.001

### Decline of physical functions

**Figure 6:**
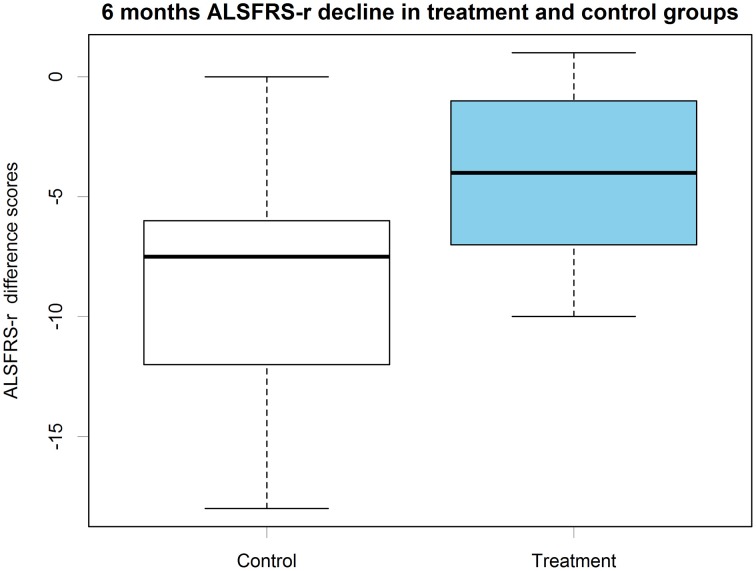
**ALSFRS-r 6 months difference scores boxplots in control and treatment groups**.

**Figure 7:**
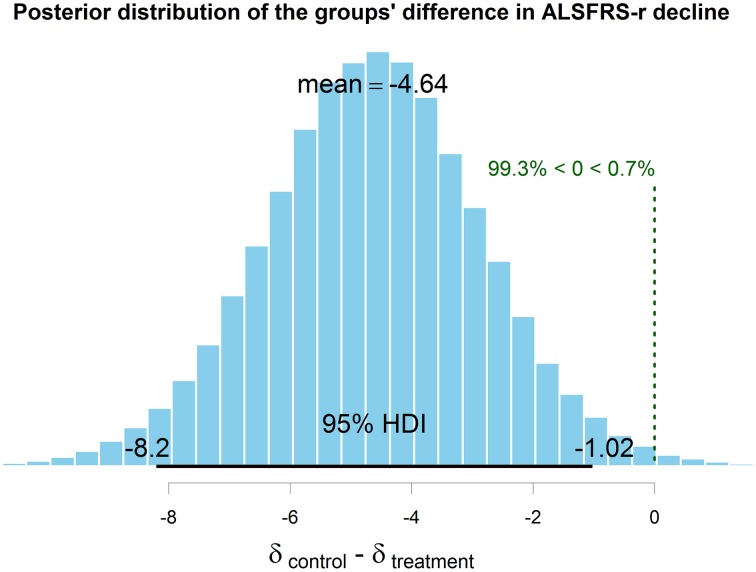
**The computed posterior distribution of the true difference in means between experimental and control group decline at 6 months**. Since zero lies outside the 95% HDI (the interval that contains the exact value with the most credibility), it can be inferred that the two groups' decline at 6 months is credibly different.

For both the patients' groups we computed ALSFRS-r difference scores between pre-treatment and the 6 month's follow up (Figure 6). Treatment (*M* = −3.98, *SD* = 3.49) and control group's (*M* = −8.64, *SD* = 4.80) scores were compared through Bayesian estimation. The computed posterior for the difference in groups' means showed a credible average difference of −4.63 with 95% HDI (−8.213, −1.02), which means a 99.3% probability of the real difference value to be smaller than zero (Figure 7). The computed Bayes Factor against the “no difference” null model was 6.70, which indicates a substantial evidence in favor of the “difference” model.

Furthermore, we run Bayesian linear mixed-models regression analysis on the treatment group to assess if the decline of the ALSFRS-r score could be predicted by our psychological measures or other factors. The best fitting model, though, showed no effect aside the decline in time (BF against “Id only” model = 7036, *R*^2^ = 0.95, RSE = 2.01, model parameters are reported in Table 4).

**Table 4:** **Posterior parameters for ALS Functional Rating Scale-revised best model**.

**Parameter**	**Mean**	***SD***	***SE***
Intercept	31.642	2.697	0.008
T0	1.358	0.441	0.002
T1	1.362	0.441	0.002
T2	−0.481	0.429	0.001
T3	−2.239	0.476	0.002
Sigma^2^	4.025	0.956	0.005

Perceived severity of secondary symptoms, as qualitatively collected in clinical interviews, were compared between pre and post treatment levels: sleep disorders improved in 4 out of 5 patients, pain and cramps improved in 11 out of 14 patients, fasciculations improved in 8 out of 12 patients and emotional lability improved in all of the 7 patients presenting such symptom.

### Psychological wellbeing of caregivers

#### Anxiety

**Figure 8:**
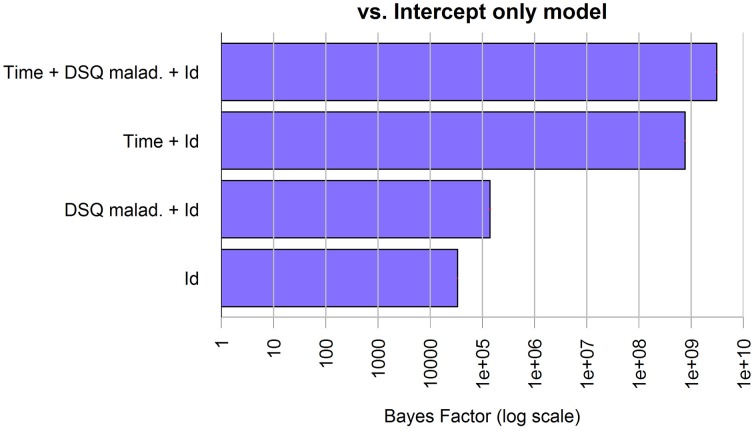
**Bayes Factors of the chosen model for anxiety in caregivers and its simpler nested models against the null model**. The bars, on logarithmic scale, represents the ratio of evidence between the different models. Thus, by visual comparison, the weight of each single factor can be inferred.

The best model selected through the Bayesian linear regression showed an effect of the treatment (Time factor) and the DSQ maladaptive score on caregivers' anxiety, but no interaction factors. The model shows an overall good fit (BF against “Id only” model > 1000, *R*^2^ = 0.79, RSE = 1.90, model parameters are reported in Table 5), additional BF are reported in Figure 8.

**Table 5:** **Posterior parameters for HADS-Anxiety best model in caregivers**.

**Parameter**	**Mean**	***SD***	***SE***
Intercept	6.711	0.831	0.003
T0	2.106	0.449	0.002
T1	0.928	0.428	0.001
T2	−1.381	0.447	0.002
T3	−1.653	0.451	0.002
DSQ–maladaptive	−1.406	0.792	0.004
Sigma^2^	3.606	0.902	0.005

#### Depression

**Figure 9:**
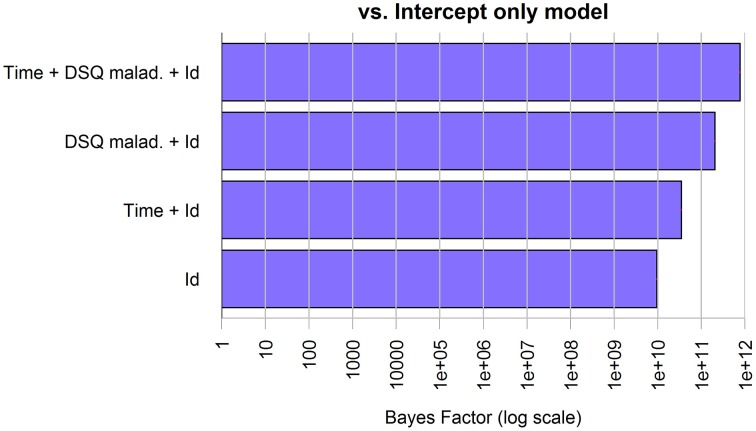
**Bayes Factors of the chosen model for depression in caregivers and its simpler nested models against the null model**. The bars, on logarithmic scale, represents the ratio of evidence between the different models. Thus, by visual comparison, the weight of each single factor can be inferred.

The best model selected through the Bayesian linear regression showed an effect of the DSQ maladaptive score and of the treatment (Time factor) on caregivers' depression, but no interaction factors. The model shows an overall good fit (BF against “Id only” model > 1000, *R*^2^ = 0.79, RSE = 1.90, model parameters are reported in Table 6), additional BF are reported in Figure 9.

**Table 6:** **Posterior parameters for HADS-Depression best model in caregivers**.

**Parameter**	**Mean**	***SD***	***SE***
Intercept	3.619	0.711	0.002
T0	0.787	0.362	0.001
T1	0.246	0.330	0.001
T2	−0.411	0.345	0.001
T3	−0.622	0.357	0.001
DSQ–maladaptive	−2.065	0.748	0.004
Sigma^2^	2.623	0.639	0.004

## Discussion

The primary goal of the present study was to provide evidence on the efficacy of a hypnosis-based intervention on patients with ALS. Confirming our hypotheses, the results of this longitudinal investigation showed an improvement in wellbeing of patients with ALS, essentially in line with preliminary data observed in our pilot study (Palmieri et al., 2013). Namely, after our hypnotic treatment and autohypnosis training, we observed an improvement in anxiety and depression scores, as measured by HADS (Zigmond and Snaith, 1983). Regression analysis clearly described the improvement in anxiety score, persisting also after 3 and 6 months, while depression score returned to the pre-treatment level at 3 months not allowing for any of the considered factors to describe satisfyingly its longitudinal trend.

With regard to QoL, as measured by ALSSQOL-r (Felgoise et al., 2009) total score, our best possible regression model described an evident effect of intervention, although of only moderate extent. Specifically we observed an improvement at the end of the treatment and after 3 months, while at the 6-months follow-up the scores turned slightly back toward baseline levels. ALSSQOL-r's individual subscales showed the same trend, except for the “intimacy” subscale, which improved only at 3 months and did not regress at the last follow-up; the “bulbar” subscale, which showed a constant decline; and the “interaction” subscale, which already returned to baseline levels after 3 months.

Bayesian mixed-models regression analysis described no relevant effects of the mild psychopharmacological treatment assumed by five of our patients, nor of their different hypnotizability levels, as assessed by PCI (Pekala, 1991). Analogously, the constancy in the practicing of self-hypnosis after the treatment did not revealed any effect in the study outcomes.

Furthermore, our hypotheses of a link between psychological intervention and physical decline, based on previous studies on ALS describing the psychological status as a moderator for disease progression/survival rate, were confirmed. Specifically we observed an improvement in perceived secondary physical symptomatology immediately after the treatment and, notably, a slower functionality loss, comparing our treated group to a ALS control group, one-by-one matched in terms of age, gender, onset site, time from onset, ALSFRS-r (Cedarbaum et al., 1999) score at baseline and absence of any cognitive impairment. DSQ (Bond et al., 1983) questionnaire was used to assess defense mechanisms profile to individuate the role of intrapsychic factor on psychological status; the DSQ consists of three subscales corresponding to three major defense styles: image distorting, maladaptive, and adaptive, sorted by increasing adaptivity. The scores observed in our patients suggest they strongly rely on the most primitive spectrum of the defense styles. We found some peculiar defense mechanism to be much more represented than others, and overall, patients' scores in maladaptive and image distorting subscale were about the double than those reported in the normative data, while the scores for the adaptive style were comparable to those of the normal population. The treatment had a positive effect on caregivers' anxiety and depression scores as well. This effect was found to persist after 3 and 6 months from the intervention. The DSQ scores regarding the caregivers group substantially mirrored those found for patients with the exception of the “fantasy” defense mechanism, which had a very high score in the patients' group only. Additionally, the maladaptive style score was found to have an effect, constant in time, on anxiety level in patients and on anxiety and depression levels in caregivers. Notably we found no interaction effect between the improvement in time and the DSQ scores either in patients or in caregivers, meaning that the efficacy of the treatment was independent from the amount and type of defenses used.

Each of these aforementioned results, in the context of our main hypotheses, could open a very wide area of discussion, with in-depth theoretical analysis and consequent clinical applications. Among the most relevant results, the firsts that may deserve such a detailed focus are the positive effects of the treatment. Before the intervention, anxiety appeared to be the greater psychological source of suffering in the treatment group's patients, especially in comparison to depression. Specifically, the clear reduction of anxiety scores, immediately after the 1-month hypnotic treatment, was observed to persist during all our data collection period. Consistently with these results, several evidences converge in suggesting that hypnotic state and hypnotizability could be strongly moderated by oxytocin (Bryant et al., 2012; Bryant and Hung, 2013), a hormone implicated, indeed, in social bonding and in the reduction of social anxiety (Radke et al., 2013).

Before treatment, in contrast to anxiety, depression was found at “borderline” levels at most. While the scores decreased after the intervention, the observed depression level was probably too low, at every time point, to appreciate a clearly evident final effect of the treatment.

Although anxiety and depression are frequently described as two strongly related dimensions in ALS literature (Vignola et al., 2008; Atassi et al., 2011; Chen et al., 2015), their different extent measured in our study, could be interpreted in the light of the concerns raised by several authors about assessment methodologies, in particular for the evaluation of depression levels (Wicks et al., 2007; Gibbons et al., 2011; Gibbons and Young, 2012). Additionally, while undoubtedly linked, anxiety and depression may reveal different complexities regarding their clinical investigation and assessment, given their peculiar nature and pathogenesis. From a psychodynamic perspective, anxiety is typically experienced when a person feels himself to be in impending danger, whether internal (as a consequence of pulsional conflicts) or external (because of real or presumed threats). It is a common clinical experience, other than theoretical assumption, conversely, that depression is a more sophisticate and rich phenomenon, which pervades many life aspects. Furthermore, it is conceivable that the existential despair caused by ALS together with the concomitant challenge to live positively the remaining time, as described by many patients, could generate conflicting feelings poorly represented in the generic items of a standardized questionnaire. The mourning process caused by the progressive and relentless loss of patients' physical functions generates overwhelming experiences of demoralization, hopelessness, anger, and loss of meaningfulness that should not be simply ascribed to “depression” (Blackhall, 2012). Additionally, our current western culture denies and censors the thought of death, even at an institutional level, resulting for instance, in the lack of adequate end-of-life policies, formalized procedures and professionals training observed by Cipolletta and Oprandi (2014) in health care organizations. Such a widespread cultural denial may have relevant psychological consequences, such as the inability to verbally express, or even consciously conceive, the dread caused by the reality of their condition (Testoni et al., 2013). For all of these reasons some aspects of depressive symptomatology may have passed unnoticed.

QoL is as well one of the most important among the yet achievable goals in the context of ALS psychological treatment. In our results, we found that QoL, as a whole, showed a clear immediate positive trend, slightly reverting 6 months after treatment. When compared to the QoL questionnaire sub-dimensions, the intimacy subscale revealed a clear amelioration only 3 months after the treatment, and persisting for the whole observation period. This delayed effect is probably associated with the great improvement in anxiety and depression levels observed in caregivers, which surprisingly kept stable at 6 months after patients' treatment. Such a virtuous circle in which the better psychological wellbeing of the patients positively influences caregivers' one and, vice versa, caregivers' improvement shows a positive impact on patients' psychological status has already been described in some published articles (Chiò et al., 2004, 2005; Lillo et al., 2012; Pagnini et al., 2012b).

Overall, the burden experienced by the caregivers of patients with ALS is often severe and they are at risk of developing depressive and anxiety symptoms (Pagnini et al., 2010). In our opinion, although representing a secondary outcome in our experimental design, the results observed in regard to caregivers may have great relevance also in terms of clinical practice.

As for the verbal content and the administration modality, our hypnosis-based protocol based its theoretical premises on well-established literature on hypnosis, already described as effective in depressive symptomatology (Shih et al., 2009), in anxiety (Saadat et al., 2006; Nash and Barnier, 2012), in the improvement of QoL (Liossi and White, 2001) as well in the treatment of many neurological diseases conditions (Wahbeh et al., 2008). In particular, since suggestions are the core mechanisms withstanding the hypnotic treatment, it can be inferred that those specifically included in our protocol could have promoted the psychological change in patients.

Each of the four selected suggestions themes, while shaped on patients' individual features were conceived to have a role in the conscious and unconscious dynamics, in order to promote long-term benefit. For instance, thinking about the past and the future has been described to lead to increased rumination and well-being loss in ALS patients (Real et al., 2014). By means of hypnosis it is possible to intervene in this painful process, leading to a sense of hopelessness (Plahuta et al., 2002), through the vivid recall of positive imagery of the past and the projection of these symbolic representations into the future, a process which allows to restructure the experience of the self in a coherent and significant temporal dimension. This goal was pursued through the “life-chain” suggestion, based on imageries of generations of ancestors, which were represented behind each patient's own shoulders and the patients' offspring and future generations in front of them. These imageries, while evoking a feeling of hope, triggers patients' support functions, ideas of protection and help, and therefore may reactivate secure attachment figures.

In this vain, many of our hypnotic inspirations included in the suggestions also refer to the attachment theory, in terms of relationships which provide secure-base and safe-haven functions. Clear and stable decrement of patients' anxiety levels, which is one of the main outcome observed in our study, could have partially been due to the symbolic representation of sources of protection conceived in the attachment theory framework, as suggested by Mikulincer et al. (2010).

Another relevant finding of our study is the attenuation of the disease progression, after 6 months, in the treated patients when compared to a well-matched ALS control group. As the number of participants was relatively small, this potentially very relevant finding should be considered cautiously, and requires further investigations to be confirmed. However, this result would be supported by those previous studies showing how a poor psychological state is a significant predictor of subsequent disability and can influence survival rate (McDonald et al., 1994; Johnston et al., 1999; Krampe et al., 2008; Pagnini et al., 2014a). This could be a broader mechanism, not only affecting the person health-related choices, such as advance health care directive for LTMV, as observed by Rabkin et al. (2006).

The association found between the attenuation in disease progression and the concomitant psychological treatment can have several interpretations. It is a fact that the central nervous system changes during hypnosis have been documented by fMRI and EEG studies, and there are many sites in the nervous system in which the overall neural output to skeletal muscle can be modified (Faymonville et al., 2006; Raz et al., 2006). Specifically, hypnosis can have effects on physical parameters such as pain threshold, inflammatory states (for an exhaustive review see Jensen et al., 2015), and, according to Rossi and colleagues, also at a genomic level (Rossi, 2003).

Indeed, increasing evidence shows that psychophysiological processes, as well as psychotherapy, have an impact on the brain morpho-functional arrangement and genic expression (Benelli et al., 2012; Morath et al., 2014).

However, many questions remain regarding the mechanism that explain the effect of hypnosis in our study. Possibly, the specific muscular relaxation suggestions included in our protocol, could have played a role, independently from the hypnotic phenomenon *per se*, in training patients not to overload, and thus to partially preserve, the still innerved muscular fibers.

While the hypnotic intervention proved efficacious in all treated patients, we did not find any relevant effect of the PCI's HSS index, measuring the individual ability to reach a deeper trance and strongly correlated to classic hypnosis measures, such as the Harvard group scale of hypnotic susceptibility (Pekala and Kumar, 1984, 1987; Forbes and Pekala, 1993), not suitable in a clinical context. Although this result may appear counter-intuitive, in the context of hypnotic research it is not as surprising. The exact relationships between hypnosis components (e.g., hypnotizability, suggestibility, dissociation, relaxation, expectancy, compliance, etc.) are not yet well understood, and high hypnotizability was not found to be necessary, nor predictive for treatment efficacy in other hypnotic intervention contexts as well (Carli et al., 2008).

Finally, particular attention has been devoted to individuate the identification of the defense mechanisms employed by patients, which inevitably modulate the ways the disease is faced, and consequently the psychological reactions. Our data showed an impressive strengthening of the primitive organizational level of defense mechanisms, included in the “maladaptive” and “image-distorting” styles; e.g., projection, regression, projective identification, denial, omnipotence, etc.

A sort of “well-being paradox,” meaning that a person shows an apparently good psychological health in the face of an incurable disease, has been observed in terminal patients (Grehl et al., 2011). This phenomenon could be explained by the massive use of denial (i.e., a repudiation of a painfully shared reality and the replacement of it with a more agreeable, individual, version), classically described also in ALS patients (Brown and Mueller, 1970).

Weisman provides an interesting distinction between three aspects of denial in terminal illness (Weisman, 1972). A “first order” denial, in which patients deny the facts relative to his illness; a second-order denial, which prevents the patients from evaluating the extent and the implication of the disease; and a third order denial directed to the image of death itself. In this sense, denial mechanisms could be interpreted more in an “adaptive,” rather than “primitive” perspective, in a context that obliges patients to face their own mortality.

Preconceived notions regarding this frequently described defense mechanism in ALS, such as “denial is good” or “denial is bad” do not make sense in the clinical situation. Indeed, denial could serve the patient well if it wards them off anxiety or depression without interfering with treatment compliance or the patient's life goals (Straker, 1998). However, albeit denial was intensively used, the most employed defense mechanism observed in our sample was projection (i.e., attributing to others tendencies originally located and identified within oneself, and then condemned and disavowed), in particular in terms of paranoid thinking. Items related to the idea of other people posing a threat to the patients' own physical and psychical life, received extremely high scores (all patients scored 9 or 10, on a 10 points Likert scale), probably related to that painful feeling of “punishment” wittily detected in clinical interviews with patients by Oster and Pagnini (2012).

Lastly, we observed a strong reliance on the escapist “fantasy” defense mechanism, already observed in patients with ALS by Palmieri et al. (2010a) by means of projective measures. Results of our regression analyses showed that the maladaptive style score was the only factor, aside from treatment, that played a significant role in moderating psychopathological symptoms. Specifically acting as a buffer against the manifestation of anxiety and depression, an effect that was constant in time and active in both patients (although statistically sound only for anxiety) and their caregivers, who showed a very similar pattern of defense mechanisms. These data suggests that the presence of projection and denial, as well as other archaic defense mechanisms, could actually reveal an adaptive reaction to such devastating conditions, and allow to better welcome further treatment as opposed as to what usually happens in clinical psychological practice with patients without physical diseases (McWilliams, 2006).

Among the limitations of the study, the paucity of the sample may restrict the generalizability of the study findings, yet some characteristic of our protocol design represents innovative strength points in respect to the small literature on ALS psychological treatment. Firstly, the patients assessed in the present study were screened for cognitive dysfunctions. This is a relevant issue in the disease (Abrahams, 2013), which is almost never considered in ALS psychological treatment, and is an aspect that may potentially mask the severity and multi-facetedness of psychopathology in these patients. Furthermore, the chosen hypnotic treatment, together with self-hypnosis training, allows for another important goal in the context of such a pervasive disease as other psychological interventions, such as cognitive therapy, require an interactive conversation, which becomes eventually almost impossible as the disease progress toward a locked-in state (Kurt et al., 2007). Self-hypnosis instead is a skill that, once learned, can be reliably used without the need of any muscular activity. In psychodynamic terms, entering a state of self-hypnosis taught in a therapeutic context, allows to reactivate an introjected representation of the therapist as well, together with its associated supporting meanings of a safe relationship.

In synthesis, we highlight the longitudinal, positive effects of our brief psychodynamic treatment, both at psychological level, and most strikingly, at physical level. Our data are similar, for some aspects, to those recently described by Averill et al. (2013); Díaz et al. (2014); Pagnini et al. (2014b), and their teams for treatments, respectively based on expressive disclosure approach, cognitive-behavioral therapy, and mindfulness meditation training. Still, to the best of our knowledge, this is the first report of a positive long-term effect on disease's progression associated to a psychological intervention in these patients.

On a theoretical level, we tried to approach the complexity of the psychological dynamics in reaction to a devastating disease such ALS, not by searching just for a common personality or typical psychological patterns. We attempted instead to highlight individual peculiarities and integrate them in every design step: in the treatment, in data analysis, by controlling the subject factor, and finally in the results interpretation, moving first steps toward the goal of abductive reasoning (Salvatore and Valsiner, 2010). The abductive generalization (Peirce, 1935), that could represent the key to overcome the opposition between nomothetic and idiographic perspectives, is not only compatible, but arguably strictly interdependent with Bayesian confirmation theory (Douven, 2011) on which the statistical methods employed in our study are based, and we hope that these dialog and perspective could represent the future directions in this research field.

In the clinical practice perspective, patients and caregivers should be aware that hypnotic interventions conjunctly to training in self-hypnosis are relatively easy and inexpensive to provide, and have a plethora of beneficial “side effects.” We hope our findings could represents a helpful step in the construction of “best clinical practice,” to better manage psychic and physical plagues imposed by ALS disease to patients and their families.

### Conflict of interest statement

The authors declare that the research was conducted in the absence of any commercial or financial relationships that could be construed as a potential conflict of interest.
